# The mediating role of nutritional care literacy on the relationship between self-directed learning ability and nursing competence

**DOI:** 10.1186/s12912-024-02185-4

**Published:** 2024-07-29

**Authors:** Yanhong Peng, Lingling Tan, Ke Zhang, Na Zhu, Hongjian Dong, Hong Gao

**Affiliations:** 1 Department of Nursing, The Second Affiliated Hospital, Hengyang Medical School, University of South China, Hengyang, China; 2 School of Nursing, University of South China, Hengyang, China

**Keywords:** Self-directed learning ability, Nutritional care literacy, Nursing competence, Mediation effect, Clinical nurses

## Abstract

**Background:**

Nurses' nursing competence and nutritional care literacy directly affect patients' health and safety. Self-directed learning ability was pervasive throughout the entire work process of nursing work and was the basis for improving both. However, there are few studies has explored the mechanism from the perspective of nutritional care literacy. The purpose of this study was to analyze the relationship between self-directed learning ability and nursing competence, and to explore the mediating role of nutritional care literacy between self-directed learning and nursing competence among clinical nurses in China.

**Methods:**

A cross-sectional survey was conducted among 805 clinical nurses recruited from seven general hospitals in Hunan Province, China, between January 25 and March 6, 2022. The self-directed learning ability, nutritional care literacy and nursing competence of nurses were evaluated through investigation. A total of 799 questionnaires were received, resulting in an response rate of 99.25%.We performed an intermediary modeling to examine the mediating roles of nutritional care literacy on the relationship between self-directed learning ability and nursing competence in clinical nurses.

**Results:**

Self-directed learning ability was positively correlated with nutritional care  literacy (*r*=0.792, *P*<0.001) and nursing competence (*r*=0.696, *P*<0.001). Nutritional care literacy was positively correlated with nursing competence (*r*=0.658, *P*<0.001). Nutritional care literacy mediated the relationship between self-directed learning ability and nursing competence. The mediating effect accounted for 32.48% of the total effect and 48.10% of the direct effect .

**Conclusions:**

This study confirmed the positive correlation between self-directed learning ability, nutritional care literacy, and nursing competence. Nutritional care  literacy played a mediating role in the relationship between self-directed learning ability and nursing competence. The findings not only provide a novel strategy for cultivating nursing professionals and improving nurse disease care abilities, but also offer a new perspective for nursing educators and managers.

## Introduction

Nursing is a profession that requires the constant updating of knowledge and skills as well as lifelong learning [1]. Nursing staff must possess strong self-directed learning abilities to adapt to the development of the nursing discipline and changes in health care systems more effectively [1]. Self-directed learning ability refers to the comprehensive capacity of nurses to proactively assess their learning needs, formulate precise learning objectives and strategies, and evaluate the outcomes of their learning endeavors [2, 3]. This ability simultaneously represents a significant manifestation of nurses' comprehensive qualities and constitutes an integral component of evaluations of continuing education and training in the field of nursing [4]. According to the purpose of learning, nurses self-directed learning mainly by looking up information on the Internet, participating in online and offline training and reading books [5]. The effect of self-directed is closely related to the learning environment and learning style [2]. Nursing competence has been included in Quality and Safety Education for Nurses (QSEN) [6]. This notion is utilized to evaluate the learning outcomes, competence and proficiency of nurses [7]. Effective self-directed learning ability promotes the continuous improvement of personal nursing competence [4]. The cultivation of nursing competence not only formal education but also nurses' continuous and effective learning in clinical practice to enhance their application of knowledge [8]. It directly impacts the well-being and safety of patients [9]. Nursing competence encompasses the care abilities of nurses in the clinical field, outstanding humanistic care ability in humanistic nursing, and the abilities to achieve career goals and effectively manage stress [7]. However, the enhancement of nursing knowledge and skills, the achievement of career goals, and stress management are all intricately intertwined with learning. Studies have shown a negative association between self-directed learning ability and stress as well as a positive association between self-directed learning ability and professional identit [10, 11]. Moreover, self-directed learning ability is pervasive throughout the entire work process and serves as a fundamental pillar of lifelong learning [12]. Possessing strong self-directed learning ability facilitates the assimilation of novel skills, knowledge, tools, and ideas within one's professional domain [13]. Therefore, it can be posited that self-directed learning ability serves as the foundation for the enhancement of nursing competence and may contribute to its improvement.

Health literacy refers to an individual's ability to access and understand essential health information and services, as well as make informed decisions based on such information and services [14]. Previous study demonstrated that health literacy was a key mediating factor in the relationship between medical staff support and the patients’ prognosis [15]. Health literacy facilitated active communication between patients and healthcare providers [16]. One study found that a direct relationship between health literacy and nursing competence, suggesting that improving nursing staff's health literacy effectively enhanced their nursing competence [17]. But most health literacy studies does not explicitly focus on nutritional [18]. Nutritional care literacy refers to the comprehensive literacy that nurses possess with regard to conducting accurate nutritional risk screenings, dietary assessments, and nutritional evaluations for patients as well as effectively implementing dietary guidance and providing appropriate nutritional support [19]. It is an important factor in improving health literacy. Nutrition support therapy has emerged as a prominent clinical intervention, while nutritional care has become an integral facet of nursing practice [20]. Nurses in internal medicine, surgery, pediatrics, obstetrics and gynecology, and other departments should possess nutritional care literacy to effectively implement nutritional programs [21]. They play a crucial role in both nutritional screening and the dissemination of nutrition science [21]. Nutrition knowledge, skills, assessment, health education and other qualities all contribute to the professionalism of nursing [22], thereby impacting nursing competence. In summary, nutritional care literacy potentially serving as a predictive factor with regard to nursing competence. Nurse-led patient health education is crucial for promoting patients self-care management [23]. Nursing managers should develop nutrition care literacy training programs to improve nurses' competency of nutrition management and health education.

Research findings have indicated a general deficiency in nutrition care knowledge among nurses [24, 25]. The insufficiency of nutrition knowledge may impede the effectiveness and comprehensiveness of dietary education, thus hindering the improvement of patients' dietary behavior and nutritional status [26]. The nutritional care literacy of nurses is related to their professional knowledge of nutrition, nutritional evaluation literacy, health education literacy and corresponding quality [27]. Nurses can improve their nutritional care literacy by actively engaging in continuous and effective learning, as well as summarizing within their work. Nurses who possess a high level of self-directed learning ability can effectively utilize various learning pathways and information resources, formulate learning strategies, and successfully acquire nutrition-related knowledge and skills [28]. Therefore, it can be inferred that the acquisition of self-directed learning ability constitutes a crucial factor in the promotion of literacy in nutritional care.

**Fig. 1:**
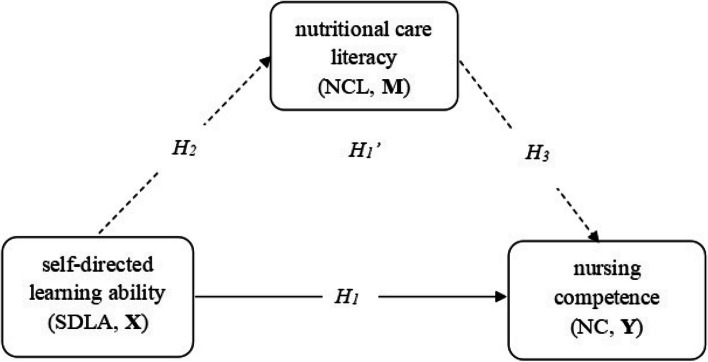
Research hypothesis framework of mediation model

In summary, self-directed learning ability promoted the improvement of nursing competence, and nutritional care literacy might be a predictor of nursing competence. Additionally, self-directed learning ability contributesd to the improvement of nutritional care literacy. However, the relationship among these three factors remains unclear. The present study collected data using scales to assess self-directed learning ability, nutritional care literacy, and nursing competence among clinical nurses from seven general hospitals in Hunan Province. The aim of this study was to explore the relationship between self-directed learning ability and nursing competence, and to determine whether nutritional care literacy serves as a potential mediating factor in this relationship (refer to Fig. 1). It can provides evidence for the development of continuing education programs and enhancement of nursing competence.The following hypotheses were proposed in this study.

### Research hypotheses

**H1:** Self-directed learning ability positively influences nursing competence.

**H2:** Self-directed learning ability positively influences nutritional care literacy.

**H3:** Nutritional care literacy positively influences nursing competence.

**H1':** Nutritional care literacy positively mediates the relationship between self-directed learning ability and nursing competence.

## Methods

### Participants and data collection

The study adopted a convenient sampling method and a descriptive-analytical research design. A cross-sectional survey was conducted among 805 clinical nurses recruited from seven general hospitals in Hunan Province, China, between January 25 and March 6, 2022. The inclusion criteria for this study focused on nurses with a minimum of 1 year of clinical experience. The exclusion criteria encompassed individuals who dropped out from the study or who took sick leave or maternity leave during the survey period. Participants only included if they completed all aspects of the questionnaire. A total of 799 questionnaires were received, resulting in an response rate of 99.25%. The present study employed an electronic questionnaire, which was administered online to participants in a confidential manner. The web-based questionnaire star platform was employed to distribute the online questionnaires among clinical nurses(https://www.wjx.cn/). Participants were provided with electronic informed consent and their explicit consent was obtained. A comprehensive explanation of the study's objectives and detailed instructions for completing the questionnaire were provided to participants. The first page of the questionnaire contained an introduction to the primary survey contents and emphasized adherence to the principle of confidentiality. The initial section of this questionnaire was self-administered and focused on demographic information, including gender, age, years of professional experience, job title, hospital level and department affiliation. The sampled nurses were offered the right to withdraw from the study at any point. All data were checked by two researchers.

### Sample size

According to the principles of international questionnaire design, reliability results can be obtained when the sample size is 5–10 times larger than the number of entries in the questionnaire [29]. The total number of questionnaire items in this study was 124, including 34 items assessing self-directed learning ability among nurses, 63 items measuring nutritional care literacy using a scale designed for clinical nurses, and 27 items evaluating nursing competence. Given the incompleteness and inefficiencies of the questionnaire, it was recommended to augment the sample size of this study by 10%. Therefore, the minimum necessary sample size was 682, as determined by the analysis conducted.

### Measurement scales

#### The self-directed learning ability of nurses

The scale was used to measure the self-directed learning ability for nurse**s.** The scale developed by Xiao SQ and Li XH in china [8] and featured a Cronbach’s alpha coefficient of 0.944 and a Kaiser–Meyer–Olkin value of 0.934. Cronbach's alpha estimates reported in this study was 0.993. The reliability and validity information that were collected and reported gathered for the Chinese scale. This scale contained a total of 34 items across 4 dimensions: self-learning motivation, task analysis, self-monitoring and regulation, and self-evaluation. A 5-point Likert scale was used for each dimension (ranging from 1 = completely inconsistent to 5 = completely consistent), with a total score from 34 to 170. The total score was calculated as the aggregate of individual item scores, with higher values indicating a greater level of self-directed learning ability.

#### The nutritional care literacy scale for clinical nurses

The nutritional care literacy scale for clinical nurses was developed by Zhu et al. [27] in China and utilized to assess the nutritional care literacy of clinical nurses. The Cronbach’s alpha coefficient for this scale was 0.991, indicating high internal consistency reliability, and the retest reliability coefficient was 0.876, suggesting good stability over time. Cronbach’s alpha of the scale in this research was 0.994. The scale contained 63 items across 5 dimensions, including nutrition professional knowledge, literacy in evaluating clinical nutrition, nutrition health education literacy, nutrition support care literacy, and nutrition care-related quality. This measure was scored on a 5-point Likert scale ranging from 1 (completely inconsistent) to 5 (completely consistent) for each item. The total scores range from 63 to 315, with higher values indicating a greater level of nutritional care literacy.

#### Nursing competence instrument

The scale was developed by Lin et al. [7], translated and adapted to the Chinese context by Yang et al. [30]. The scale was used to assess the nutritional nursing literacy of nurses. The internal consistency of this scale, as measured by Cronbach's alpha, was found to be 0.909. Moreover, the retest reliability coefficient was calculated to be 0.835. Cronbach's alpha in this study was 0.988. The questionnaire contained a total of 27 items across 4 dimensions, including integrating care abilities, guiding humanity concerns, improved career development and dealing with stress. The scale was scored on a 10-point Likert scale ranging from 1 (strongly disagree) to 10 (strongly agree), and the total scores range from 27 to 270, with higher scores indicating greater competence.

### Statistical analysis

The data entry and management tasks were performed using Excel software, while data analysis was conducted using IBM SPSS 23.0 software. Descriptive analysis was used for quantitative data, which is presented as the mean ± standard deviation. Categorical variables are described in terms of frequency and percentage. Pearson correlation analysis was used to identify the correlations among self-directed learning ability, nutritional care literacy, and nursing competence. The mediating effects of nutritional care literacy could be explored when a statistically significant Pearson correlation was observed between variables in the hypothesized direction [31]. A *p* value ≤ 0.05 was considered to be statistically significant. The PROCESS macro version 4.1(Model 4) was used to estimate the mediation model. The 5000 bootstrap samples was to measure the indirect effects. The established mediating (indirect) effect were considered to be statistically significant (*P* < 0.05), if the 95% bias-corrected confidence interval results excluding zero [32]. The mediation analysis was controlled for work experience, age, professional title, and clinical department.

### Ethical approval

The present study was granted ethical approval by the ethics committee of the second affiliated hospital, Hengyang Medical School, University of South China, Hengyang, China (No.2022-0120-09). Prior to participation in the investigation, all respondents provided informed consent. Respondents voluntarily and anonymously participated in this study.

## Results

### Demographic characteristics

A total of 799 clinical nurses, who were aged 18 to 54 years (mean age: 32.64±6.75) and whose work experience ranged from 1 to 36 years (mean (11.11±7.14), were included in the study. The majority of participants were female (98.2%, *n*=785), although several males were included (1.8%, *n*=14). Among participants, 627 nurses had attained a university degree or higher level of education (78.5%). A total of 805 participants were recruited from seven general hospitals located in Hunan Province, China, between January 25 and March 6, 2022, using a convenience sampling method. All 799 questionnaires were received, resulting in an response rate of 99.25% (799/805).The demographic characteristics of the respondents are presented in Table 1.

**Table 1:** Demographic characteristics of the respondents (*N* = 799)

Variables	Categories	Frequency (n)	Percentage (%)
Sex	Female	785	98.2
	Male	14	1.8
Education background	Senior high school	9	1.1
	Junior college	163	20.4
	Undergraduate or above	627	78.5
Professional title	Junior nurses	473	59.2
	Intermediate nurses	283	35.4
	Senior nurses	43	5.4
Hospital level	Class-A Grade-3 general hospital	603	75.5
	Class-A Grade-2 general hospital	196	24.5
Clinical department	Internal Medicine	253	31.7
	Surgery	181	22.6
	Emergency and Critical care	79	9.9
	Pediatric	43	5.4
	Obstetrics and Gynecology	30	3.8
	outpatient	76	9.5
	operating room	50	6.2
	Other	87	10.9

Others include: Radiology Department, Medical Laboratory Science, etc

### Correlations among nutritional care literacy, self-directed learning ability, and nursing competence

The scores for self-directed learning ability, nutritional care literacy,and nursing competence among nursing were 133.88 ± 25.56, 235.07 ± 48.09, 217.75 ± 41.72, respectively. Correlation analysis was conducted to identify the potential correlations among the three variables. Nutritional care literacy (r = 0.792, *P*<0.001) and nursing competence (*r*=0.696,* P*<0.001) were significantly positively correlated with self-directed learning ability. Nutritional care literacy was positively correlated with nursing competence (*r*=0.658, *P*<0.001). The results regarding the Pearson correlation coefficients and descriptive analyses are shown in Table 2.

**Table 2:** Correlations and descriptive analyses

Variables	Mean	SD	SDLA	NCL	NC
SDLA	133.88	25.56	1		
NCL	235.07	48.09	0.792**	1	
NC	217.75	41.72	0.696**	0.658**	1

**P < 0.001

*Abbreviations*: *SD *Standard deviation, *SDLA *Self-directed learning ability, *NCL *Nutritional care literacy, *NC *Nursing competence

### Mediation effect model

**Fig. 2:**
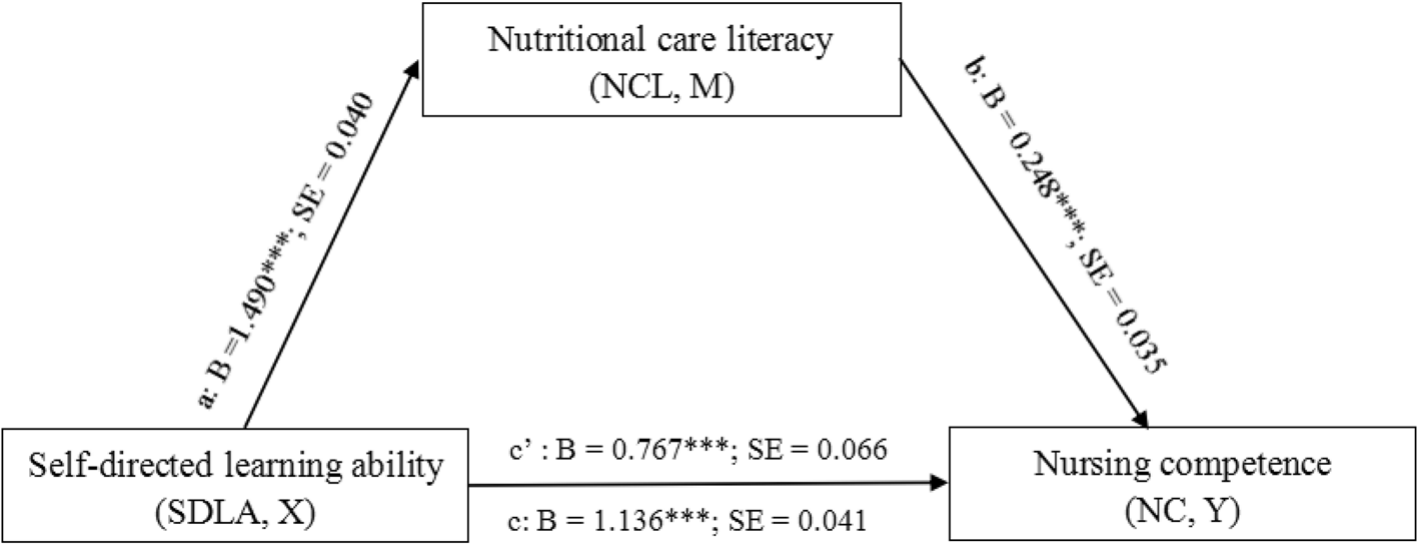
Mediation model of how does the self-directed learning ability influences the nursing competence identity via nutritional care literacy. a=direct effect of X on mediator M; b=direct effect of mediator M on Y; c=total effect of X on Y; c*’*=direct effect of X on Y; ***P < 0.001

Based on correlations and preliminary results, nutritional care literacy was investigated as a potential mediator of the association between self-directed learning ability and nursing competence through Model 4 in PROCESS. Work experience, age, professional title, and clinical department set as covariates. The results showed that self-directed learning ability had a significant effect on nurses’ nutritional care literacy (*B *= 1.490, *SE* = 0.040, *t *= 36.615, *P *< 0.001) and nursing competence (*B *= 0.767, *SE* = 0.066, *t *= 11.628, *P *< 0.001), nutritional care literacy signifcantly positively predicted nursing competence (*B *= 0.248, *SE* = 0.035, *t *= 7.074, *P *< 0.001). The mediating effect of the nutritional care literacy of clinical nurses was found to be statistically significant (*P*< 0.001), as the 95% CI did not include zero (Table 3). The mediating effect accounted for 32.48% of the total effect and 48.10% of the direct effect. The mediation model and path coefficients are shown in Figure 2. It is worth noting that the mediation model was statistically signifcant (*F *= 423.166, *P *< 0.001), the R^2^ and R values were 0.515 and 0.718 respectively.

**Table 3:** Total, direct, and indirect effect of mediation analysis

	Effect	SE	95% LLCI	95% ULCI
Total effect of X on Y	1.136	0.041	1.055	1.218
Direct effect of X on Y	0.767	0.066	0.637	0.896
Indirect effect (X→M→Y)	0.369	0.059	0.255	0.487

*X *self-directed learning ability, *M *nutritional care literacy, *Y *nursing competence

## Discussion

This study investigated the association between self-directed learning ability and nursing care competence among clinical nurses in China, with a focus on investigating whether nutrition literacy acts as an intermediary variable. Our findings revealed that the self-learning ability of clinical nurses was significantly positively correlated with nutritional care literacy and nursing competence. Nutrition care literacy plays a partial mediating role in this relationship. The findings is crucial for educators and nursing managers to gain a comprehensive understand of the pathway towards cultivating nursing talents, promoting the reform of nursing education curriculum, and perfecting the clinical practice training program.

The results showed that the self-directed learning ability of nurses was at an intermediate level and requires further improvement. It was basically consistent with the study of Tan et al. [1]. This study demonstrated that self-directed learning ability was positively correlated with nursing competence. The finding, consistent with previous research results, demonstrated that self-directed learning ability of nursing students had a direct positive effect on their clinical competencies [33]. Similarly, the positive correlation between nursing students' self-directed learning ability and their critical thinking skills established by previous research also supported our research results [13]. Lee et al. [34] also discovered that nursing students' self-directed learning ability has a significant positive impact on their professional nursing values. Advanced levels of self-directed learning can foster the development of good professional values, thus empowering individuals to effectively fulfill their responsibilities. In addition, Hwang et al. [35] arrived at a similar conclusion, highlighting a significant positive correlation between self-directed learning and problem-solving skills among nurses. The self-directed learning facilitates nurses' engagement in in-service training, enhances their vocational performance, nursing competence and professional skills [36]. Guide nursing managers in developing appropriate training programs based on nurses' varying self-directed learning abilities, aiming to enhance learning effects and address the challenges arising from ongoing social and technological developments within the health care industry [37].

The study conducted by Li et al. [38] demonstrated that the acquisition of nutrition knowledge through learning formal nutrition courses and regularly attending  nutrition training programs significantly contributed to enhancing clinical nurses' nutritional care literacy. Nutrition training not only enables medical staff to better identify and deal with nutrition problems, but also stimulates their interest in nutrition knowledge [39]. Furthermore, other research results have emphasized the importance of nurses' nutritional knowledge and literacy for the successful implementation of nutritional therapy and nutritional health education [40, 41]. The foundation of improvement nutritional care literacy lies in the ability to continuously learn and access information [12]. This study also revealed a significant positive correlation between self-directed learning ability and nutritional care literacy. Therefore, nursing educators should focus on cultivating independent learning abilities and offering a series of courses on nutrition knowledge, assessment, health education, and nutrition nursing skills to improve learning efficiency.

The results indicated that nutritional care literacy positively influenced nursing competence, which was consistent with the findings of Jung et al. [15] and Li et al [17]. Hence, enhancing nurses’ nutritional care literacy can inherently improve their overall nursing aptitude. Some studies have highlighted the pivotal role of specialized nurses in nutrition within multidisciplinary treatment teams, including comprehensive nutritional assessment and screening, personalized dietary requirements determination, the provision of nutritional support, and the delivery of informative counseling and health education services[42, 43]. All of these efforts have contributed significantly to the improvement of patient recovery and overall health care outcomes. Moreover, research has demonstrated that nutritional care interventions administered by specialist nurses in nutrition is effective with regard to reducing the incidence of nosocomial infection, decreasing hospital stays, improving patients' clinical prognoses, decreasing readmission rates, and lowering nursing costs [44–46]. Therefore, nurses must acquire pertinent knowledge to proficiently evaluate dietary intake and provide appropriate nutritional guidance and counseling to patients [47]. These also contribute to the career development of nurses.

The findings of this research demonstrated that nutritional care literacy played a  partially mediating role in the relationship between self-directed learning ability and nursing competence. The discovery that self-directed learning among nursing students was positively associated with their career development also provided empirical support for this finding [48]. The results showed that the better self-directed learning ability of nurses, the higher nutritional care literacy. Nurses with high nutritional care literacy are good at summarizing new learning paths and models in health education and nutrition management and other work, thereby improving their self-directed learning ability. At the same time, nutritional care literacy is conducive to improving disease management capabilities, thereby improving nursing competence and promoting patient health. The relevant standards mandate that nurses at all levels should provide patients with nutrition-related nursing interventions, thus emphasizing the importance of continuous learning by nurses to enhance their knowledge and contribute to the provision of high-quality nursing care [49]. Therefore, nursing competence should be improved by enhancing self-directed learning ability and strengthening nutritional care literacy.

### Limitations

In addition, these findings contribute to enriching both the theoretical framework and practical application of self-directed learning and nutrition education-related research within the nursing profession. However, this study had several limitations. First, this study was cross-sectional study, which enabled us only to establish clear associations among variables rather than to elucidate the causal relationships among them. Second, self-reported measures were employed to assess self-directed learning ability, nutritional care literacy and nursing competence. However, this approach may have introduced response bias, as clinical nurses could have overestimated their abilities. This limitation should be considered when interpreting the findings. Third, this study collected data solely from clinical nurses drawn from seven general hospitals in Hunan Province. Hence, the generalization of the conclusions of this research to other cultural backgrounds and geographic ares may be limited, and these findings cannot represent the condition of all clinical nurses. Therefore, more empirical evidence is required to strengthen the results of this study through future research.

## Conclusions

Self-directed learning ability was found to have positive impacts on both nutritional care literacy and nursing competence. Additionally, nutritional care literacy played a mediating role in relationship between self-directed learning ability and nursing competence. The overall enhancement of self-directed learning ability and nutritional care literacy was conducive to improving nursing competence. These findings offered a pragmatic perspective for educators and nursing administrators on the efficacious role of self-directed learning in enhancing nursing competence, including nutritional care literacy. It is recommended that educators incorporate content related to self-directed learning ability into the nursing education curriculum. Nursing managers should consider organizing in-service training or continuing education activities to improve nurses' nutritional care literacy, thereby optimizing patient care.

## Data Availability

The datasets used during the current study are available from the corresponding author on reasonable request.
